# Physical exercise and children’s resilience: mediating roles of self-efficacy and emotional intelligence

**DOI:** 10.3389/fpsyg.2025.1491262

**Published:** 2025-03-21

**Authors:** Caixia Jiang, Kun Wang, Hao Qin

**Affiliations:** ^1^Konggang Jiayuan Primary School, Chongqing, China; ^2^College of Physical Education, Southwest University, Chongqing, China; ^3^Rose City South Experimental Primary School, Chongqing, China

**Keywords:** physical exercise, children, self-efficacy, emotional intelligence, resilience, chain mediating

## Abstract

**Purpose:**

This study aims to examine the inner relationship between children’s physical exercise, self-efficacy, emotional intelligence, and resilience, and explore effective ways to promote the improvement of children’s resilience.

**Methods:**

Using measurement tools such as the Physical Activity Rating Scale (PARS-3), the General Self-Efficacy Scale (GSES), the Chinese version of the Emotional Intelligence Scale (EIS), and the Adolescents Resilience Scale, we conducted a questionnaire survey was conducted among 700 primary school students in 4 primary schools. The SPSS 22.0 software was used to process and analyze the data, including correlation analysis, regression analysis, and Bootstrap analysis, and AMOS 21.0 software was used to establish a structural equation model.

**Results:**

(1) Physical exercise has a significant positive correlation with self-efficacy (*r* = 0.29, *p* < 0.001), emotional intelligence (*r* = 0.32, *p* < 0.001), and resilience (*r* = 0.37, *p* < 0.001), and there is also a significant positive correlation between emotional intelligence and resilience (*r* = 0.30, *p* < 0.001). (2) Physical exercise can directly and positively predict children’s resilience (β1 = 0.38) and its five sub-dimensions, that is, physical exercise can positively predict goal concentration (*β* = 0.35, *p* < 0.001), emotional control (*β* = 0.29, *p* < 0.001), positive cognition (*β* = 0.20, *p* < 0.01), family support (*β* = 0.33, *p* < 0.001), interpersonal assistance (*β* = 0.31, *p* < 0.001), respectively. (3) Self-efficacy (SE = 0.10) and emotional intelligence (SE = 0.08) have partial mediating effects, respectively, on the relationship between physical exercise and resilience, and the chain mediation effect of self-efficacy and emotional intelligence also reaches a significant level (SE = 0.02).

**Conclusion:**

Physical exercise can have a positive impact on children’s resilience through self-efficacy and emotional intelligence, so schools or parents should attach importance to children’s physical activities, which is an effective way to improve their resilience.

## 1 Introduction

Resilience, also known as psychological elasticity, psychological resilience, or ability to face adversity, is an individual’s ability or trait to cope with stress, setbacks, or trauma. It is an important positive psychological quality (Davydov et al., 2010; Windle, 2011). It is closely related to the mental health of children (senior primary school students) (Li et al., 2022). Typically, individuals with high resilience have richer psychological resources and the ability to flexibly mobilize internal and external resources to cope with difficulties. They are more likely to gain growth opportunities from negative events and turn adverse factors into motivation (Pietrzak et al., 2009; Troy and Mauss, 2011; Xi et al., 2011). Research has found that resilience is a protective factor for individual mental health and can reduce the negative impact of life events on mental health in a wide range (Ye et al., 2014; Han et al., 2019). Senior primary school students (9–12 years old) are in the critical period of transition from childhood to adolescence. They are facing major changes in physiology, psychology, and interpersonal relationships, and are more likely to develop negative emotions such as depression and anxiety (Yang, 2012). As one of the important psychological resources, an increase in resilience will allow individuals to gain more satisfaction and develop better life resources (Abolghasemia and Taklavi, 2010), thereby having more internal and external resources to cope with stressful events, have a stronger ability to deal with negative emotions, thereby improving life satisfaction (Tugade and Fredrickson, 2004). Therefore, examining the protective factors of children’s resilience and the relationship paths between variables from the perspective of positive psychology, and then finding effective ways to improve children’s resilience, is the key to promoting their mental health development.

### 1.1 The relationship between physical exercise and resilience

With the development of exercise psychology, the positive relationship between physical exercise and individual resilience has received widespread attention. For example, researchers have found that children who regularly participate in physical activities have higher resilience, and high resilience can improve their adaptability and academic performance (Granizo et al., 2019). Individuals with high resilience are usually emotionally stable, have strong social adaptability and emotional management capabilities, and can better focus on goals when facing tasks. Even if they encounter negative events in study or life, they are more likely to respond with a positive attitude (Dong et al., 2023). Research shows that extracurricular physical exercise can promote children’s mental qualities of self-confidence and tenacity (Yan et al., 2019), and positively predict children’s resilience (Li et al., 2022). For example, football training has a significant effect on improving the resilience of senior primary school students (Liu, 2017). Meanwhile, the positive relationship between physical exercise and resilience has been effectively confirmed in college students (Liu, 2020) and children with attention deficit hyperactivity disorder (ADHD) (Liang et al., 2023). It can be explained as physical exercise is conducive to enhancing blood circulation and hormone secretion, promoting metabolism, effectively helping the body relax and divert attention, and thus has a positive effect on improving resilience (Deng et al., 2025; Li et al., 2024). It can be seen that the development of children’s resilience is important for healthy growth, but there are relatively few studies on the relationship between physical exercise and children’s resilience, and whether this relationship is affected by other variables is still unclear.

### 1.2 The relationship between physical exercise, self-efficacy, and resilience

Self-efficacy refers to the subjective perception of an individual’s belief in whether he or she can complete a certain behavior or task at a certain level (Bandura, 1986). It is people’s evaluation of their abilities (Bandura, 1982), which is the psychological power of individuals’ continuous self-regulation, which can predict and explain corresponding behaviors (Bandura, 1997). Research shows that there is a significant correlation between physical exercise and self-efficacy (Tang, 2000). Among children and adolescents, the correlation between physical exercise and self-efficacy has been confirmed by some studies (Zhao et al., 2019) , and the increase in physical activity, is more conducive to improving the performance of adolescents’ self-efficacy (Zhao et al., 2019). In other words, physical exercise plays an important role in improving an individual’s self-efficacy, and self-efficacy will undergo adaptive changes as the degree of exercise increases (Downs and Strachan, 2016). For example, an individual’s self-efficacy can be positively altered after an intense run or bike ride (McAuley et al., 1995). Meanwhile, children with high self-efficacy usually have a higher ability to cope with pressure, frustration, or trauma, that is, there is also a significant positive correlation between self-efficacy and children’s resilience (Liang, 2011). Children with high resilience have significantly higher scores on general self-efficacy than children with low resilience, indicating that the former have higher confidence in their ability to cope with pressure or challenges (Hamill, 2003; Xi et al., 2011). Research shows that individuals with high self-efficacy have higher behavioral motivations, can set practical goals for themselves, evaluate themselves more positively when facing difficulties, and are more able to use positive coping methods to solve the problems they face (Schwarzer and Warner, 2013), which helps individuals grow from difficulties and promotes the development of resilience (Feng et al., 2022). Further research found that self-efficacy has a mediating effect between physical exercise and mental health (Sheng et al., 2016). There is a mutual relationship between physical exercise, self-efficacy, and resilience among college students, and physical exercise can have a positive effect on resilience by promoting self-efficacy (Chen, 2023).

### 1.3 The relationship between physical exercise, emotional intelligence, and resilience

Emotional intelligence refers to the ability to express, evaluate, and regulate one’s own and others’ emotions, as well as the ability to use emotions to solve practical problems, that is, a comprehensive ability to accurately perceive, evaluate, and express emotions (Salovey and Mayer, 1990; Mayer and Salovey, 1997). Research has found that emotional intelligence has an important impact on children’s interpersonal adaptation, academic achievement, and social adaptation (Zhang, 2014; Meng, 2018). Children with good emotional intelligence can express, manage, and reflect on their problems well. Emotions, thereby stimulating the ability to think positively, act positively, and coordinate interpersonal relationships (Yang, 2010). In recent years, the relationship between physical exercise and the emotional intelligence of students at different stages has received widespread attention from researchers. For example, Zhu (2020) found that moderate-intensity physical exercise at different times can improve the emotional intelligence level of primary school students, and especially has a significant improvement effect on primary school students’ self-emotion regulation and emotional perception. Ruiz-Ariza et al. (2019) found through research that cooperative high-intensity intermittent exercise has a significant impact on the creativity and emotional intelligence of middle school students, and can effectively improve the creativity, happiness, and social abilities of inactive middle school students. Wang et al. (2020) found through a cross-sectional survey that there is a positive correlation between physical exercise and emotional intelligence, and regular participation in physical exercise can promote the improvement of emotional intelligence among college students. Interestingly, the improvement of emotional intelligence can create conditions for the development of children’s resilience and can be used as a protective factor for resilience. For example, in stressful situations, emotion regulation ability and positive emotional experience serve as protective factors and are conducive to enhancing individual resilience (Olsson et al., 2003). A study on college students pointed out that emotional intelligence is positively related to resilience. Students with outstanding emotional intelligence tend to have higher levels of resilience, which is related to the ability to effectively regulate emotional information (Fa and Wang, 2021). The results suggest that physical exercise is related to the improvement of individual emotional intelligence (Ubago-Jimenez et al., 2019), and individuals with high emotional intelligence can better perceive, regulate, and control information about themselves and their surrounding environment (Higgs and Dulewicz, 2014), and good self-emotional assessment, emotional regulation, and emotional utilization can effectively predict resilience (Wen et al., 2014a).

### 1.4 The chain mediating effect of self-efficacy and emotional intelligence

Previous studies have shown that children’s physical exercise is related to self-efficacy (Huang et al., 2018; Isa et al., 2019; Xue et al., 2023), self-efficacy, and resilience (Hamill, 2003; Liang, 2011), and there is a significant correlation between physical exercise, self-efficacy and resilience (Chen, 2023). Meanwhile, physical exercise is related to emotional intelligence (Ruiz-Ariza et al., 2019; Zhu, 2020), emotional intelligence, and resilience (Liu et al., 2020), and there are different degrees of physical exercise, emotional intelligence, and resilience correlated (Wen et al., 2014a; Ubago-Jimenez et al., 2019). Interestingly, research has found that the general self-efficacy level of adolescents is related to their emotion regulation ability. The higher the self-efficacy level, the stronger their emotion regulation ability (Liu et al., 2011). Physical exercise can not only directly improve students’ negative emotional levels, but also indirectly improve students’ negative emotional levels by improving students’ self-efficacy (Liu, 2020).

To sum up, there are varying degrees of relationships between physical exercise, self-efficacy, emotional intelligence, and resilience. However, previous studies have mostly explored pairwise correlations between variables and lacked an overall perspective to examine the interrelationships between variables, thus limiting the generalizability and theoretical depth of the conclusions. In addition, psychological resilience is a comprehensive psychological index that involves many influencing factors. The path relationship between different factors has not been effectively verified through the model, and relevant research on children is rare. Therefore, this study intends to construct a chain mediation model between physical exercise, self-efficacy, emotional intelligence, and resilience, aiming to reveal the internal correlation between the variables and explain the protective factors of psychological resilience, to find ways to improve Effective paths for children’s resilience and mental health. Therefore, by constructing a chain mediation model among physical exercise, self-efficacy, emotional intelligence, and resilience, this study aims to reveal the internal correlation among various variables and explain the protective factors of resilience, to find an effective path to improve children’s resilience. Based on this, the following hypothesis was proposed in this study: H1: Physical exercise can positively predict children’s resilience. H2: Self-efficacy has a mediating effect between physical exercise and resilience. H3: Emotional intelligence has a mediating effect between physical exercise and resilience. H4: Self-efficacy and emotional intelligence have a chain mediating effect between physical exercise and children’s resilience.

## 2 Participants and methods

### 2.1 Participants

This study adopted a cross-sectional survey research design and randomly selected 4 primary schools (Yubei District Airport Jiayuan Primary School, Yubei Central Park Primary School, Yubei Luozhi Primary School, and Yubei Bashu Primary School) in Yubei District, Chongqing, China to conduct a questionnaire survey. The samples involved students in the fourth, fifth, and sixth grades of primary school. Inclusion and exclusion criteria for sample selection: (1) No physical disability or movement disorder; (2) No mental illness or psychological disorder. In addition, based on previous research and to facilitate the establishment of a good structural equation model, we distributed a total of 700 questionnaires, recovered 655 questionnaires, eliminated 35 invalid questionnaires, and finally obtained 620 valid questionnaires. Among them, there are 277 boys and 343 girls. The average age of the participants is 9.99 ± 1.02 years old, the height is 1.48 ± 0.09 m, and the weight is 40.56 ± 12.30 kg. Questionnaire collection procedure: To ensure the quality and reliability of filling in the questionnaire, after explaining the precautions and requirements for filling out the questionnaire in detail to the students, participants should complete the filling in the questionnaire individually within 20 min according to their actual situation and collect it on the spot. In addition, to facilitate the participants to fill in the questionnaire, the distribution and filling of the questionnaire were carried out in the classroom during the break time. Written informed consent was obtained from all participants for this study. Meanwhile, since the survey targets a group of minors, we also obtained the approval of the parents of the participants in advance and signed the parent-informed consent form. This study was approved by the Ethics Committee of the School of Southwest University, China (SWU-TY202105) and followed the Declaration of Helsinki, and written informed consent was obtained from all participants.

### 2.2 Research methods

#### 2.2.1 Scale design

##### 2.2.1.1 Physical activity rating scale (PARS-3)

The “Physical Activity Rating Scale” (PARS-3) used was compiled and revised by Liang (1994), which evaluates subjects’ physical exercise from three aspects: exercise intensity, exercise frequency, and exercise time. It is quantified using a 5-point Likert type, with scores ranging from 1 to 5, based on which the level of participation in physical exercise is measured. Physical exercise score = exercise intensity score × (exercise time score - 1) × exercise frequency score. The score range is 0–100 points. The higher the score, the greater the amount of physical exercise. In this study, the test–retest reliability of this scale was high, with a correlation coefficient of r = 0.82. The factor loading is greater than 0.5, the AVE is greater than 0.6, and the combined reliability CR is greater than 0.6, indicating that this scale has good convergent validity. Meanwhile, the Cronbach alpha coefficient of this scale is 0.78. The measurement validity and reliability of this scale are shown in Table 1.

**Table 1:** Reliability and validity test results of each scale.

Scale	KMO and Bartlett sphericity test	Sub-dimension	Items	Characteristic root	Explanatory variance (%)	Progressive explanatory variance (%)	Cronbach *α*
PARS-3	——	——	3	1.79	59.62	59.62	0.78
EIS	KMO = 0.92,	Emotion perception	11	8.39	47.68	47.68	0.89
	*p* < 0.001	Self-emotion management	6	1.78	8.43	56.11	0.81
		Other people’s emotion management	10	1.45	6.52	62.63	0.91
		Emotion application	6	1.06	5.35	67.98	0.84
Measurement model validation results		x^2^/df = 1.85, RMSEA = 0.03, AGFI = 0.98，TLI = 0.98, CFI = 0.93，IFI = 0.99, GFI = 0.97					
GSES	——	——	10	1.93	60.18	60.18	0.85
ARS	KMO = 0.95,	Goal focus	5	9.06	46.77	46.77	0.85
	*p* < 0.001	Emotional control	6	1.93	8.12	54.89	0.77
		Positive cognition	4	1.68	7.65	62.54	0.82
		Family support	6	1.35	6.41	68.95	0.80
		Interpersonal assistance	6	1.10	5.19	74.14	0.86
Measurement model validation results		x^2^/df = 2.01, RMSEA = 0.03, AGFI = 0.97, TLI = 0.99, CFI = 0.95, IFI = 0.96, GFI = 0.98					

##### 2.2.1.2 Chinese version of the emotional intelligence scale (EIS)

The emotional intelligence scale was revised by Wang and He (2002) was used. This scale has a total of 33 items, of which 5, 28, and 33 are reverse-scored. It contains four dimensions: emotion perception (11 items), self-emotion management (6 items), other people’s emotion management (10 items), and emotion application (6 items). A 5-point Likert scale is used for quantification. The items “very inconsistent, relatively inconsistent, unclear, relatively consistent, and very consistent” are scored as 1–5 points, respectively. The score range is 33–165 points. The higher the score, the higher the emotion. The stronger the intelligence. In this study, the test–retest reliability of this scale was high, with a correlation coefficient of r = 0.80. Each factor loading is greater than 0.7, the AVE is greater than 0.6, and the combined reliability CR is greater than 0.6, indicating that this scale has good convergent validity. The measurement validity and reliability of this scale are shown in Table 1.

##### 2.2.1.3 General self-efficacy scale (GSES)

The general self-efficacy scale was revised by Wang et al. (2001) was used. This scale is a unidimensional scale with a total of 10 questions. A 5-point Likert scale was used for quantification. The items “disagree, disagree very much, generally, somewhat agree, strongly agree” were scored as 1–5 points, respectively. The score range was 10–50 points. The higher the score, the higher the score, the greater the sense of self-efficacy. The stronger. In this study, the test–retest reliability of this scale was high, with a correlation coefficient of *r* = 0.83. The factor loading is greater than 0.5, the AVE is greater than 0.5, and the combined reliability CR is greater than 0.7, indicating that this scale has good convergent validity. The measurement validity and reliability of this scale are shown in Table 1.

##### 2.2.1.4 Adolescents resilience scale (ARS)

The “Adolescents Resilience Scale” compiled by Hu and Gan (2008) was used. This scale has a total of 27 items, including 5 dimensions: goal focus (5 items), emotional control (6 items), positive cognition (4 items), family support (6 items), and interpersonal assistance (6 items). The scale is quantified using a 5-point Likert scale. The items “completely inconsistent, somewhat inconsistent, unclear, somewhat consistent, completely consistent” are scored as 1–5 points respectively, with a score range of 27–135 points. The higher it is, the better the resilience is. In this study, the test–retest reliability of this scale was high, with a correlation coefficient of r = 0.85. Each factor loading is greater than 0.7, the AVE is greater than 0.7, and the combined reliability CR is greater than 0.6, indicating that this scale has good convergent validity. The measurement validity and reliability of this scale are shown in Table 1.

#### 2.2.2 Statistical methods

This study used SPSS25.0 to process and analyze the data, and used factor analysis, internal consistency testing, etc. to examine the reliability and validity of the scale; Pearson correlation analysis was used to examine the correlation coefficients between variables; Hierarchical regression analysis was used to examine the direct impact of physical exercise on emotional intelligence; Finally, Bootstrap analysis and AMOS21.0 software were used to establish a structural equation model to examine the relationship between variables and the mediating effect of self-efficacy and resilience. The significance level of all indicators was set at *p* < 0.05.

## 3 Research results

### 3.1 Common method deviation test

This study adopted a questionnaire survey method, and all questionnaire items were filled in by the subjects themselves, so there may be common method bias in the measurement. To minimize the impact of common method bias on the results, this study used anonymous questionnaire measurement, standardized testing, and other procedural control methods to control it accordingly. After the data collection was completed, this study also used Harman’s single-factor test to examine the problem of common method bias (Podsakoff et al., 2003). Put the measures of all variables together for unrotated factor analysis, the results show that there are 7 factors with characteristic roots greater than 1, and the variation explained by the first factor is 28.43%, which is less than the critical standard of 40%. As can be seen, common method bias did not pose a serious problem in this study.

### 3.2 Correlation analysis between physical exercise, self-efficacy, emotional intelligence, and resilience

Pearson correlation analysis showed (Table 2) that in this study, children’s physical exercise was related to self-efficacy (*r* = 0.29, *p* < 0.001), emotional intelligence (*r* = 0.32, *p* < 0.001), and resilience (*r* = 0.37, *p* < 0.001) all have significant positive correlations. Meanwhile, self-efficacy has a significant positive correlation with emotional intelligence (*r* = 0.31, *p* < 0.001) and resilience (*r* = 0.35, *p* < 0.001) respectively, and there is also a significant positive correlation between emotional intelligence and resilience (*r* = 0.30, *p* < 0.001). The correlation coefficients between the main research variables all reach significant levels, which provides a good basis for subsequent testing of mediating effects.

**Table 2:** Correlation between physical exercise, self-efficacy, resilience, and emotional intelligence.

Variable	M ± SD	Physical exercise	Self-efficacy	Emotional intelligence	Resilience
Physical exercise	24.89 ± 16.79	1			
Self-efficacy	25.42 ± 11.07	0.29***	1		
Emotional intelligence	62.54 ± 12.80	0.32***	0.31***	1	
Resilience	55.68 ± 11.29	0.37***	0.35***	0.30***	1

*** Means *p* < 0.001.

### 3.3 The impact and path relationship of physical exercise on children’s resilience

#### 3.3.1 Direct effect analysis

After controlling for demographic variables (gender and age), physical exercise can significantly affect the five sub-dimensions of children’s psychological resilience, as shown that physical exercise can positively predict goal focus (*β* = 0.35, *p* < 0.001), emotion control (*β* = 0.29, *p* < 0.001), positive cognition (*β* = 0.20, *p* < 0.01), family support (*β* = 0.33, *p* < 0.001), and interpersonal assistance (*β* = 0.31, *p* < 0.001), explaining 12, 8, 4, 11, and 10% of the variance, respectively (See Table 3 for details).

**Table 3:** Linear regression analysis of physical exercise on sub-dimensions of children’s psychological resilience.

Variable	Goal focus		Emotion control		Positive cognition		Family support		Interpersonal assistance	
	*β*	*t*	*β*	*t*	*β*	*t*	*β*	*t*	*β*	*t*
Gender	0.03	0.22	0.01	0.07	0.04	0.37	0.01	0.10	0.02	0.13
Age	0.08	0.67	0.06	0.46	0.06	0.44	0.03	0.19	0.10	1.01
Physical exercise	0.35***	9.28	0.29***	7.21	0.20**	5.35	0.33***	7.61	0.31***	6.90
*F*	15.78***		13.18***		8.76**		14.32***		12.47***	
*R* ^2^	0.12		0.08		0.04		0.11		0.10	

** Means *p* < 0.01, *** means *p* < 0.001.

#### 3.3.2 Mediation effect analysis

**Figure 1:**
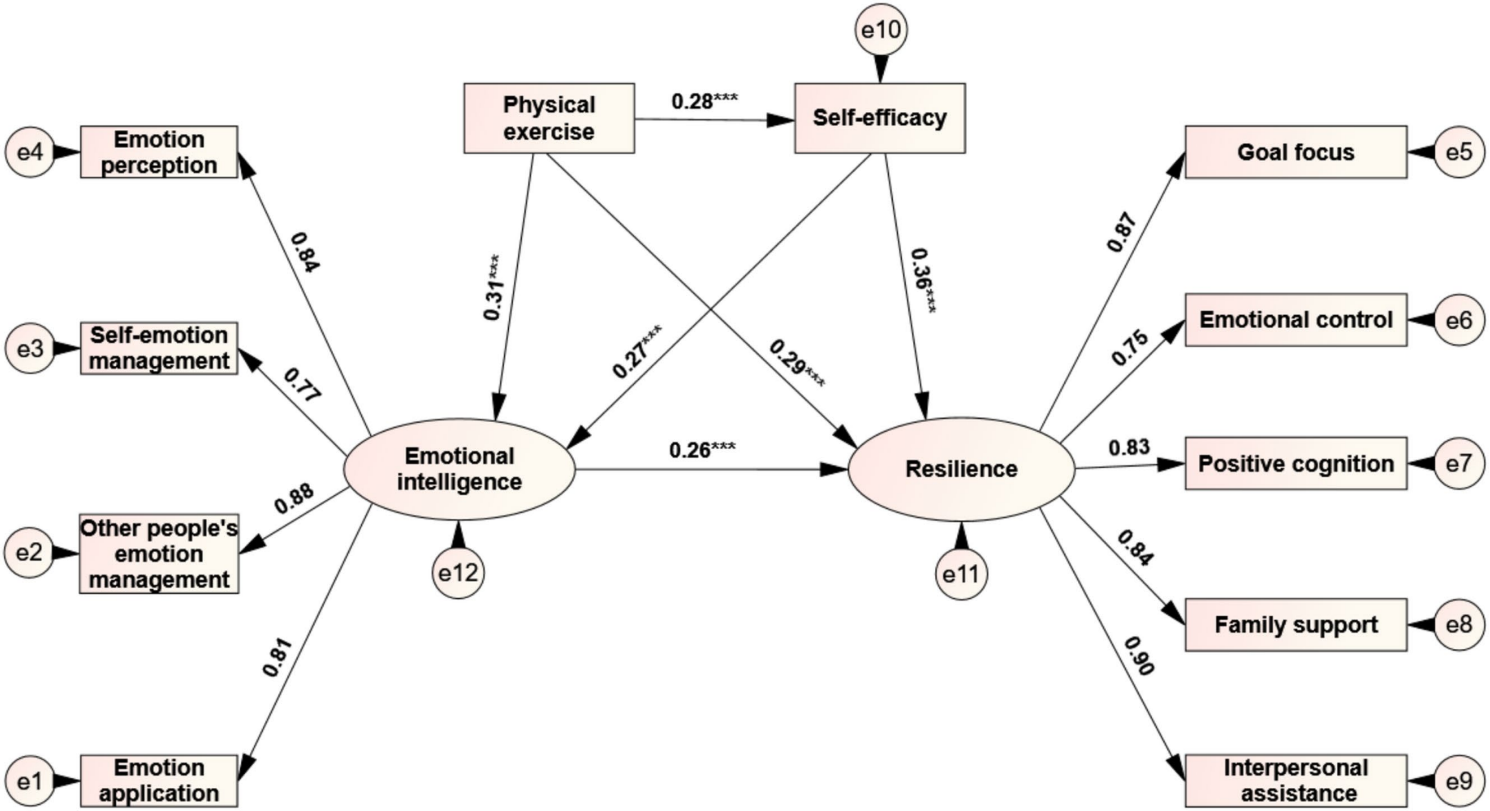
Chain mediation model between self-efficacy and emotional intelligence in physical exercise and resilience. Values in the figure are normalized regression weights.

This study followed the mediation effect test process proposed by Wen et al. (2014b) to examine the path relationship between primary school students’ physical exercise, self-efficacy, emotional intelligence, and resilience (Figure 1). After controlling for gender and age, we first tested the total effect of physical exercise on resilience and then tested the model fit and the significance of each path coefficient after adding mediating variables (self-efficacy and emotional intelligence). In the total effect model, physical exercise could directly and significantly positively predict resilience (*β*1 = 0.38, *p* < 0.001). After adding the two mediating variables of self-efficacy and emotional intelligence, physical exercise can still positively predict resilience (*β*2 = 0.29, *p* < 0.001). All fit indices of the total effect and mediation effect models reached an acceptable level (Table 4).

**Table 4:** List of fit indexes of total effect and mediating effect of physical exercise in predicting resilience.

	χ^2^/df	RMSEA	GFI	TLI	CFI	IFI	AGFI
Total effects model	2.97	0.03	0.96	0.97	0.99	0.96	0.99
Mediation effect model	2.56	0.04	0.98	0.95	0.97	0.97	0.96

The results of the mediation effect test show (Figure 1): (1) Physical exercise can significantly and positively predict self-efficacy (*β* = 0.28, *p* < 0.001), and self-efficacy can significantly and positively predict resilience (*β* = 0.36, *p* < 0.001), indicating that the mediating effect of the path of “physical exercise →self-efficacy →resilience” is significant, and its effect size is 0.28 × 0.36 = 0.10. (2) Physical exercise can significantly and positively predict emotional intelligence (*β* = 0.31, *p* < 0.001), and emotional intelligence can significantly and positively predict resilience (*β* = 0.26, *p* < 0.001), indicating that the mediating effect of the path of “physical exercise→emotional intelligence→resilience” is significant, with an effect size of 0.31 × 0.26 = 0.08. In addition, based on the research recommendations of Taylor et al. (2008) on the probability of type I error and statistical power when there is a “three-path mediation effect,” the joint significance method was used to test the chain mediation effect of “self-efficacy →emotional intelligence” between physical exercise and resilience. The results showed that: (3) self-efficacy could significantly and positively predict emotional intelligence (*β* = 0.27, *p* < 0.001), indicating that the mediating effect of the chain path of “physical exercise →self-efficacy →emotional intelligence →resilience” was significant, with an effect size of 0.28 × 0.27 × 0.26 = 0.02.

By decomposing the effect of physical exercise on children’s resilience (Table 5), it was found that the total effect of physical exercise on resilience was 0.49, the direct effect accounted for 59.18% of the total effect, and the mediating effect accounted for 40.82% of the total effect. Among them, the mediating effect of self-efficacy accounted for 20.41%, the mediating effect of emotional intelligence accounted for 16.33%, and the chain mediating effect of self-efficacy and emotional intelligence accounted for 4.08%. Each effect reached the significance level.

**Table 5:** Decomposition of the effect of physical exercise on primary school students’ emotional intelligence.

Impact path	Standardized effect size	Total effect ratio	95% Confidence interval
Direct effect	0.29	59.18%	(0.25, 0.33)
Physical exercise → Self-efficacy → Resilience	0.28 × 0.36 = 0.10	20.41%	(0.08, 0.12)
Physical exercise → Emotional intelligence → Resilience	0.31 × 0.26 = 0.08	16.33%	(0.06, 0.13)
Physical exercise → Self-efficacy → Emotional intelligence → Resilience	0.28 × 0.27 × 0.26 = 0.02	4.08%	(0.01, 0.06)
Total mediation effect	0.20	40.82%	(0.16, 0.23)
Total effect	0.29 + 0.20 = 0.49	100.00%	——

## 4 Discussion

### 4.1 The direct relationship between physical exercise and children’s psychological resilience

This study found that physical exercise has a significant positive correlation with children’s resilience (*r* = 0.37). Meanwhile, after controlling for demographic variables such as gender and age, physical exercise can directly and positively predict the five sub-dimensions of children’s resilience, and physical exercise also has a direct positive predictive effect on resilience (*β*1 = 0.38). This is consistent with previous studies, that is, physical exercise has a positive relationship with children’s resilience (Liu, 2017; Liang et al., 2023), and can positively predict children’s resilience (Li et al., 2022), which helps promote the development of psychological resilience. A review study also explained the relationship between children’s physical activity and cognitive emotions such as resilience (Cross et al., 2020). Similar conclusions have also been confirmed in the college student population, that is, physical exercise is positively correlated with the resilience of college students (Liu, 2020; Li et al., 2022). It should be pointed out that, based on previous studies, this study clarified the positive predictive effect of physical exercise on the five sub-dimensions of children’s resilience, namely goal focus (*β* = 0.35), emotional control (*β* = 0.29), positive cognition (*β* = 0.20), family support (*β* = 0.33) and interpersonal assistance (*β* = 0.31). This shows that the relationship between physical exercise and resilience is relatively complex, involving many factors such as attention, cognitive emotions, and environment. Studies have shown that physical exercise can provide an appropriate environment for improving psychological resilience (Xu et al., 2021) and that physical exercise can also benefit an individual’s psychological resilience by regulating stress perception (Arida and Teixeira-Machado, 2021). In other words, the relationship between physical exercise and resilience is diverse rather than single, and children’s active participation in physical exercise or physical activities is conducive to the overall improvement of multiple dimensions of resilience.

### 4.2 Partial mediating effect of self-efficacy between physical exercise and resilience

This study added children’s self-efficacy as a mediating variable to the mediation model between physical exercise and resilience. The results showed that self-efficacy has a partial mediating effect between physical exercise and resilience (standardized effect value SE = 0.10). The relationship between physical exercise and children’s resilience has been effectively explained above and is fully supported by previous studies (Yan et al., 2019; Granizo et al., 2019; Liang et al., 2023). Self-efficacy is an individual’s subjective evaluation of his or her ability to perform tasks (Bandura, 1982). It is closely related to behavioral choices, emotional response patterns, self-confidence, and thinking patterns. Positive self-efficacy can promote the development of abilities (Zhang et al., 1999). The amount of physical exercise is correlated with the self-efficacy of children (Huang et al., 2018) and adolescents (Zhao et al., 2019). Increasing the amount of physical exercise can help improve self-efficacy. Research shows that when individuals have strong confidence in completing tasks, they can mobilize all available resources, which will become guarantee factors for success (Tian et al., 2013) and may help improve resilience. Sabouripour et al. (2021) also believed that self-efficacy can promote the development of resilience and play a mediating role in the influence of other positive factors on resilience. Interestingly, self-efficacy plays a mediating role in the relationship between physical exercise and mental health (Jiang et al., 2018). Individuals with high self-efficacy are still able to adapt well to adversity or threats, and the goal focus, emotional control, and positive cognition of resilience play an important role (Xie et al., 2014). Meanwhile, self-efficacy also plays a mediating role between physical exercise and the mental health of middle school students. Students who participate more in sports have stronger self-efficacy, accompanied by higher subjective well-being and interpersonal adaptability (Sheng et al., 2016). Therefore, regular participation in physical exercise is closely related to the improvement of children’s self-efficacy and resilience, and physical exercise can also indirectly have a positive effect on children’s resilience through self-efficacy.

### 4.3 Partial mediating effect of emotional intelligence on the relationship between physical exercise and resilience

This study added children’s emotional intelligence as a mediating variable into the mediation model between physical exercise and resilience. The results showed that emotional intelligence has a partial mediating effect between physical exercise and resilience (SE = 0.08). Emotional intelligence is an individual’s ability to process emotional information (Mayer et al., 1999), which can guide individuals to regulate emotions reasonably (Huang and Dai, 1997) and will have a significant impact on emotional experience (Liang and Wang, 2018; Wang et al., 2020). Physical exercise plays an important role in suppressing negative emotions such as anxiety and depression in different populations and promoting the improvement of emotion regulation ability, which has been confirmed by many studies (Hayden and Allen, 1984; Xue and Zhao, 2018; Ruiz-Ariza et al., 2019; Zhu, 2020). Short-term aerobic exercise can significantly improve the executive function and emotion regulation ability of anxious students (Zhang et al., 2018). Individuals with high emotional intelligence can better perceive, regulate, and control information about themselves and their surroundings, thereby generating more positive emotional experiences (Higgs and Dulewicz, 2014). Self-emotional assessment, emotion regulation, and emotion application can better predict an individual’s psychological resilience (Wen et al., 2014a). Emotional intelligence can significantly predict individual psychological resilience (Wei et al., 2012). Emotional intelligence has a positive predictive effect on the personal strength and support of resilience (An and Pei, 2017), and is an important protective factor for resilience. On this basis, this study clarified the close relationship between physical exercise, emotional intelligence, and resilience, and revealed the partial mediating effect of emotional intelligence between physical exercise and children’s resilience.

### 4.4 Chain mediation effect between self-efficacy and emotional intelligence

To further reveal the intrinsic relationship between the variables, this study examined the chain-mediating effect of self-efficacy and emotional intelligence between physical exercise and resilience. The results showed that the chain-mediating effect of self-efficacy and emotional intelligence was significant (SE = 0.02), that is, physical exercise can affect resilience through the chain-mediation of self-efficacy and emotional intelligence. It is well known that physical exercise is one of the effective ways to improve mental health (Fu et al., 2006). On the one hand, the relationship between physical exercise, self-efficacy, and resilience has been confirmed by previous studies (Huang et al., 2018; Jiang et al., 2018; Cross et al., 2020; Sabouripour et al., 2021), and the results of this study also showed that self-efficacy played a mediating role between children’s physical exercise and resilience; On the other hand, the relationship between physical exercise, emotional intelligence and resilience has also been confirmed by previous studies (An and Pei, 2017; Zhang et al., 2018; Gong et al., 2019; Ruiz-Ariza et al., 2019), and the results of this study also showed that emotional intelligence plays a mediating role between children’s physical exercise and resilience. Interestingly, research has found that general self-efficacy is related to emotion regulation ability, and the higher the level of self-efficacy, the stronger the emotion regulation ability. Self-efficacy plays a mediating role between physical exercise and negative emotions (Wu et al., 2022). Physical exercise can significantly improve anxiety, depression, and emotion regulation self-efficacy of students at home during the epidemic. Self-efficacy plays a mediating role between physical exercise and students’ negative emotions (Liu et al., 2020). The results suggest that physical exercise can have a positive effect on children’s resilience through the chain mediation effect of self-efficacy and emotional intelligence. However, the mediating effect of emotional intelligence is lower than that of self-efficacy, which can be explained that in the indirect process of improving children’s resilience through physical exercise, self-efficacy may play a greater role and thus produce greater benefits for mental toughness, while emotional intelligence may be interfered by other factors in this process, and thus reduce the benefits for resilience.

### 4.5 Limitations

This study aims to explore the relationship between children’s physical exercise, self-efficacy, emotional intelligence, and resilience from the perspective of exercise psychology and preliminarily clarifies the intrinsic relationship between the variables. However, since this study is a cross-sectional study and uses a questionnaire survey, the research method is relatively simple and the results are more subjective, which may have potential bias of self-reported data, and it is impossible to draw deeper relationships. Longitudinal empirical studies can be included in future studies to better explore the causal relationship between variables and to further reveal the impact of relevant potentially confounding variables such as socioeconomic status. Meanwhile, the survey subjects of this study were mainly children in Chongqing, China. Due to the small differences in the same region, the research scope was relatively limited. The samples of future studies can involve more regions, expand the scope and sample size of the samples, and improve the external validity of the research results. In addition, this study mainly examined the intrinsic relationship between physical exercise, self-efficacy, emotional intelligence, and resilience. In the future, more mediating or moderating variables can be explored to enrich the research results.

## 5 Conclusion

There is a significant positive correlation between children’s physical exercise, self-efficacy, emotional intelligence, and resilience, and the relationship between variables can be further explored by comparing different physical exercises and gender in future investigations. Meanwhile, physical exercise can have a positive impact on children’s resilience through the chain mediation effect of self-efficacy and emotional intelligence. It should be noted that whether this mental health benefit continues with age and whether it is altered by other socio-demographic changes needs direct evidence from subsequent longitudinal or follow-up studies. In general, children’s resilience is a comprehensive variable that is easily affected by many factors, and regular physical exercise is an effective strategy to improve resilience, setting up longitudinal physical exercise intervention programs may be of great value in improving children’s resilience.

## Data Availability

The original contributions presented in the study are included in the article/supplementary material, further inquiries can be directed to the corresponding author/s.
